# CD100 modulates cytotoxicity of CD8^+^ T cells in patients with acute myocardial infarction

**DOI:** 10.1186/s12865-021-00406-y

**Published:** 2021-02-16

**Authors:** Yan Li, Li Qin, Qijun Bai, Jingjing Zhang, Ruixue Chen, Kunpeng Song

**Affiliations:** Department of Cardiovascular Medicine Ward II, Zhengzhou Central Hospital Affiliated to Zhengzhou University, 16 North Tongbai Road, Zhongyuan District, Zhengzhou, 450000 Henan Province China

**Keywords:** Acute myocardial infarction, CD100, T lymphocytes, Immunoregulation

## Abstract

**Background:**

**C**D100 is an immune semaphorin family member that highly expressed on T cells, which take part in the development of acute myocardial infarction (AMI). Matrix metalloproteinases (MMPs) are important mediators for membrane-bound CD100 (mCD100) shedding from T cells to generate soluble CD100 (sCD100), which has immunoregulatory effect on T cells. The aim of this study was to investigate modulatory role of CD100 on CD8^+^ T cell activity in AMI patients.

**Methods:**

Peripheral sCD100 and MMP-2 level, as well as mCD100 level on T cells was assessed in patients with stable angina pectoris (SAP), unstable angina pectoris (UAP), and AMI. The regulatory function of MMP-2 on mCD100 shedding, sCD100 formation, and cytotoxicity of CD8^+^ T cells was analyzed in direct and indirect contact co-culture system.

**Results:**

AMI patients had higher peripheral sCD100 and lower mCD100 expression on CD8^+^ T cells in comparison with SAP, UAP, and controls. CD8^+^ T cells in AMI patients showed elevated direct cytotoxicity, enhanced cytokine production, and increased perforin/granzyme B secretion. Recombinant sCD100 stimulation promoted cytolytic function of CD8^+^ T cells in controls and AMI patients. Furthermore, AMI patients also had elevated circulating MMP-2 level. Recombinant MMP-2 stimulation induced mCD100 shedding from CD8^+^ T cells and sCD100 generation, resulting in enhancement of CD8^+^ T cell cytotoxicity in AMI patients.

**Conclusion:**

Up-regulation of MMP-2 might contribute to elevation of mCD100 shedding and sCD100 formation, leading to increased cytotoxicity CD8^+^ T cells in AMI patients.

## Background

Atherosclerosis is a lipoprotein-driven disease, which results in formation of plaque at specific sites of arterial tree through intimal inflammation, necrosis, apoptosis, fibrosis, and calcification [1]. Atherosclerosis mainly causes coronary artery diseases, including stable angina pectoris (SAP), unstable angina pectoris (UAP) and acute myocardial infarction (AMI) [2]. Both innate and adaptive immune response could promote myocardial hypoxia [3, 4], resulting in the infiltration and recruitment of immune cells and adhesion of platelets. This process accelerates the progression of SAP, leading to the development of UAP and AMI [5, 6]. Activated T cell response was elevated in UAP and AMI patients compared with in SAP patients, indicating the potential involvement of T cell immunity in acute coronary syndrome [7]. CD8^+^ T cells were also accumulated in the necrotic myocardium in AMI patients and contributed to myocardial injury [8]. A recent report by Gang et al. demonstrated that peripheral CD8^+^ T cells are activated in AMI patients [9]. However, the regulatory factors for CD8^+^ T cell activation are not completely understood in AMI patients.

CD100 is also called Sema4D, which is the first discovered immune semaphorin family member with modulatory activity in vascular and immune systems [10]. CD100 could be produced by majority of hematopoietic cells, and functions as a ligand by binding to receptors depending on the cell types, including CD72 on lymphoid tissues and plexin B1/B2 on non-lymphoid tissues [11]. Plaque marcophages and foam cells in human atheomas also express CD100 [12], which contributes to marcophages-mediated inflammation in atheroscleriosis [13]. Two forms of CD100 could be found as a membrane-bound dimer (mCD100) or as a soluble molecule (sCD100) originated via proteolytic cleavage by certain factors, especially matrix metalloproteinases (MMPs) [14, 15]. Peripheral sCD100 was increased in patients with heart failure [16, 17] and atrial fibrillation [18]. However, regulation of CD100 expression and the role of CD100 to cytotoxicity of CD8^+^ T cells were not fully elucidated in coronary artery diseases.

Previous studies have shown that MMP-2, MMP-9, and MMP-14 were important mediators for sCD100 formation and mCD100 shedding from CD8^+^ T cells [14, 15]. This process was closely related to enhancement of CD8^+^ T cell activity in patients with hepatitis B virus infection and non-small cell lung cancer (NSCLC) [14, 15]. Moreover, MMP-2 was an independent and powerful predictor of all-cause mortality of patients with acute coronary syndrome [19, 20]. Thus, we designed the following study to investigate mCD100/sCD100 imbalance and regulatory function of CD100 in SAP, UAP, and AMI patients. Firstly, mCD100 on T cells and sCD100 expression was examined. Secondly, functional characteristics of MMP-2 to mCD100 cleavage and sCD100 formation towards CD8^+^ T cells were assessed in vitro.

## Results

### Plasma sCD100 and MMP-2 level was up-regulated in AMI patients

**Fig. 1:**
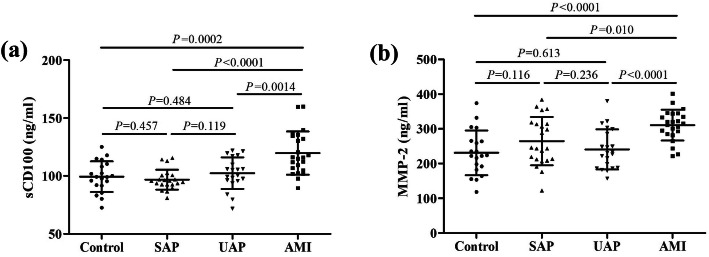
Comparison of soluble CD100 (sCD100) and matrix metalloproteinase-2 (MMP-2) level in the plasma among control group (*n* = 20), stable angina pectoris (SAP) group (*n* = 22), unstable angina pectoris (UAP) group (*n* = 20), and acute myocardial infarction (AMI) group (*n* = 23). Plasma sCD100 and MMP-2 was measured by ELISA. **a** Plasma sCD100 level was increased in AMI group than in control, SAP, and UAP group. **b** Plasma MMP-2 level was increased in AMI group than in control, SAP, and UAP group. Individual level for each subject was shown. The horizon lines indicated means, while the error bars indicated standard deviations. One-way ANOVA and SNK-*q* test was used for statistical analysis

sCD100 level was increased in AMI group (119.7 ± 18.67 ng/ml) compared with control group (99.34 ± 13.25 ng/ml), SAP group (96.78 ± 8.59 ng/ml), and UAP group (102.4 ± 13.71 ng/ml) (*P* < 0.01, Fig. 1a). Similarly, plasma MMP-2 level was also up-regulated in AMI group (310.7 ± 44.32 ng/ml) in comparison with control group (230.9 ± 64.46 ng/ml), SAP group (264.2 ± 69.54 ng/ml), and UAP group (240.7 ± 57.96 ng/ml) (*P* < 0.05, Fig. 1b).

### mCD100 on CD8^+^ T cells was down-regulated in AMI patients

**Fig. 2:**
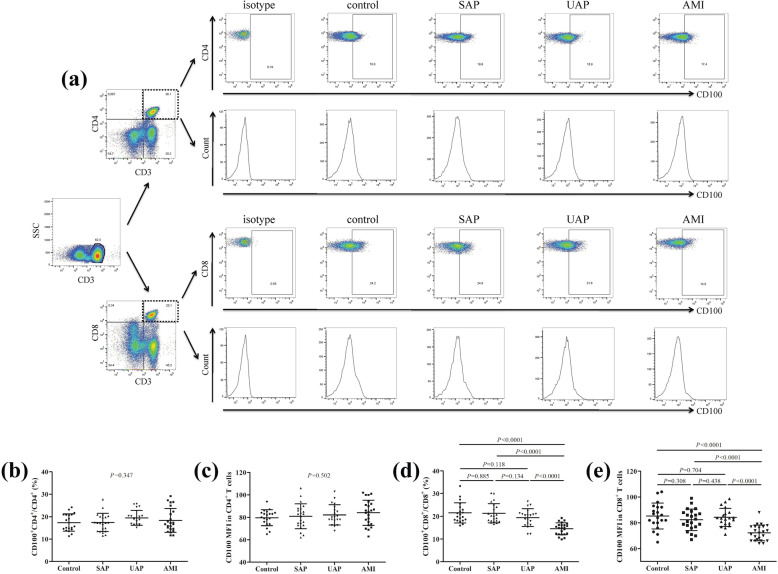
Comparison of membrane bound CD100 (mCD100) expression in CD4^+^ T cells and CD8^+^ T cells in the plasma among control group (*n* = 20), stable angina pectoris (SAP) group (*n* = 22), unstable angina pectoris (UAP) group (*n* = 20), and acute myocardial infarction (AMI) group (*n* = 23). **a** PBMCs were stained by anti-CD3, CD4, CD8, and CD100, and the representative flow dots and histograms of CD100 expression in CD4^+^ T cells and in CD8^+^ T cells in control, SAP, UAP, and AMI group were shown. The isotype control for CD100 positive and negative cells was also shown. **b** Comparison of CD100^+^CD4^+^ cells percentage among control, SAP, UAP, and AMI group. There was no significant difference of CD100^+^CD4^+^ cells percentage among control, SAP, UAP, and AMI group. **c** Comparison of CD100 mean fluorescence intensity (MFI) in CD4^+^ T cells among control, SAP, UAP, and AMI group. There was no significant difference of CD100 MFI in CD4^+^ T cells among control, SAP, UAP, and AMI group. **d** Comparison of CD100^+^CD8^+^ cells percentage among control, SAP, UAP, and AMI group. CD100^+^CD8^+^ cells percentage was reduced in AMI group compared with control, SAP, and UAP group. **e** Comparison of CD100 MFI in CD8^+^ T cells among control, SAP, UAP, and AMI group. CD100 MFI in CD8^+^ T cells was down-regulated in AMI group compared with control, SAP, and UAP group. Individual level for each subject was shown. The horizon lines indicated means, while the error bars indicated standard deviations. One-way ANOVA and SNK-*q* test was used for statistical analysis

Representative flow dots and histograms of mCD100 expression on CD4^+^ T cells and on CD8^+^ T cells were shown in Fig. 2a. There were no significant differences in either CD100^+^CD4^+^ percentage (Fig. 2b) or CD100 mean fluorescence intensity (MFI) in CD4^+^ T cells (Fig. 2c) among groups (*P* > 0.05). However, CD100^+^CD8^+^ percentage was reduced in AMI group (14.54 ± 2.64%) compared with control group (21.51 ± 4.42%), SAP group (21.31 ± 4.17%), and UAP group (19.41 ± 3.87%) (*P* < 0.0001, Fig. 2d). CD100 MFI in CD8^+^ T cells was also decreased in AMI group (72.23 ± 6.08) compared with control group (85.25 ± 9.99), SAP group (82.34 ± 8.30), and UAP group (84.21 ± 7.01) (*P* < 0.0001, Fig. 2e).

### sCD100 promoted cytolytic activity of CD8^+^ T cells

**Fig. 3:**
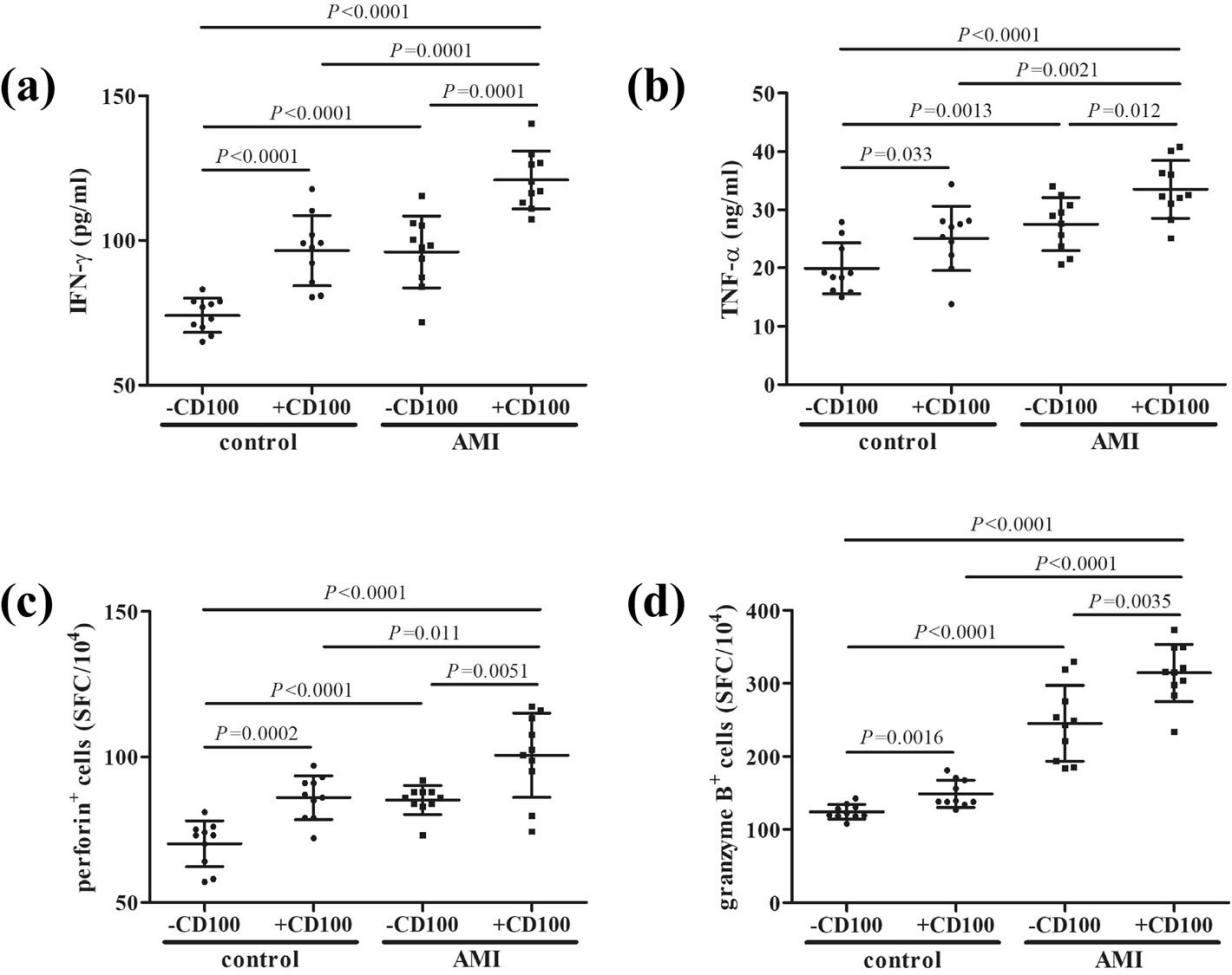
Influence of recombinant human CD100 stimulation to cytokine production and perforin/granzyme B secretion by CD8^+^ T cells from controls (*n* = 10) and acute myocardial infarction (AMI) patients (*n* = 10). CD8^+^ T cells were stimulated with recombinant human CD100 for 24 h. Cells and supernatants were then harvested. **a** IFN-γ and **b** TNF-α level in the supernatants was measured by ELISA, and was compared between controls and AMI patients, and between presence and absence of CD100 stimulation. **c** Perforin and **d** granzyme B production by CD8^+^ T cells was measured by ELISPOT. The number of spot-forming cells (SFC) was compared between controls and AMI patients, and between presence and absence of CD100 stimulation. Individual level for each subject was shown. The horizon lines indicated means, while the error bars indicated standard deviations. One-way ANOVA and SNK-*q* test was used for statistical analysis

CD8^+^ T cells, which were purified from controls (*n* = 10) and AMI patients (*n* = 10), were stimulated with recombinant human sCD100 for 24 h. The level of interferon-γ (IFN-γ) and tumor necrosis factor-α (TNF-α) was elevated in the supernatant in CD8^+^ T cells from AMI patients compared with controls (*P* < 0.01, Fig. 3a and b). The secretion of IFN-γ and TNF-α by CD8^+^ T cells was also elevated in response to CD100 stimulation in both AMI patients and controls (*P* < 0.05, Fig. 3a and b). Furthermore, the number of perforin- and granzyme B-producing CD8^+^ T cells was increased in AMI patients compared with controls (*P* < 0.0001, Fig. 3c and d). Perforin and granzyme B production by CD8^+^ T cells was increased in response to CD100 stimulation in both AMI patients and controls (*P* < 0.01, Fig. 3c and d).

**Fig. 4:**
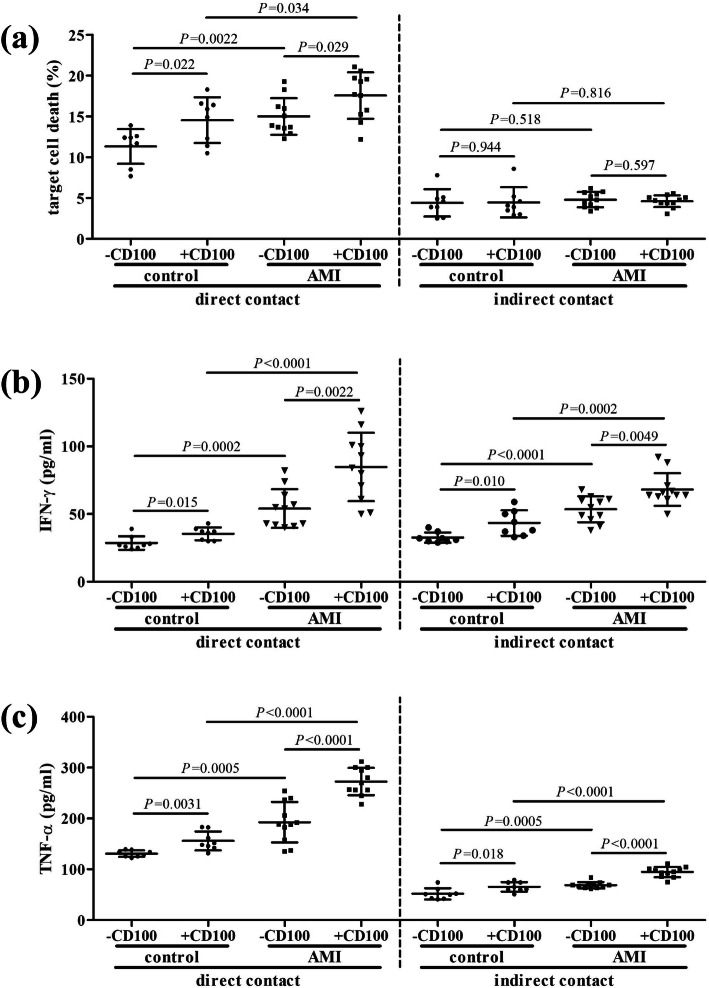
Influence of recombinant human CD100 stimulation to CD8^+^ T cells cytotoxicity from HLA-A02 restricted controls (*n* = 8) and acute myocardial infarction (AMI) patients (*n* = 10). CD8^+^ T cells were stimulated with recombinant human sCD100 for 24 h. Cells were washed twice, and 10^4^ of stimulated CD8^+^ T cells were co-cultured with 5 × 10^4^ of HUVECs in direct contact and indirect contact co-culture system. Supernatants were harvested 48 h post co-culture. **a** LDH level in the cultured supernatants were measured using LDH Cytotoxicity Assay Kit. The percentage of HUVECs death was calculated, and was compared between controls and AMI patients, and between presence and absence of CD100 stimulation. **b** IFN-γ and **c** TNF-α level in the supernatants was assessed by ELISA, and was compared between controls and AMI patients, and between presence and absence of CD100 stimulation. Individual level for each subject was shown. The horizon lines indicated means, while the error bars indicated standard deviations. One-way ANOVA and SNK-*q* test was used for statistical analysis

CD8^+^ T cells from controls (*n* = 8) and AMI patients (*n* = 11), who were HLA-A02 restricted, were stimulated with sCD100 for 24 h. CD8^+^ T cells were then washed twice, and were co-cultured with human umbilical vein endothelial cells (HUVECs) in direct contact or in indirect contact manner. Supernatants were harvested 48 h post co-culture. In direct contact co-culture manner, HUVECs death was mediated by secreting cytokines by CD8^+^ T cells and perforin-granzyme pathway, which required direct cell-to-cell contact [9]. The percentage of CD8^+^ T cell-induced HUVECs death was higher in AMI patients compared with controls (*P* = 0.0022, Fig. 4a). The percentage of HUVECs death was increased in response to sCD100 stimulation in both AMI patients and controls (*P* < 0.05, Fig. 4a), while the percentage of HUVECs death was still higher in AMI patients than in controls with sCD100 stimulation (*P* = 0.034, Fig. 4a). In indirect contact co-culture manner, HUVECs death was only mediated by CD8^+^ T cell-secreting cytokines and did not required direct cell-to-cell contact [9]. The percentage of HUVECs death induced by CD8^+^ T cells was comparable between AMI patients and controls in indirect contact co-culture manner (*P* = 0.518, Fig. 4a). Importantly, there was no significant difference of cytokine-induced HUVECs death between AMI patients and controls in response to sCD100 stimulation in indirect contact co-culture manner (*P* > 0.05, Fig. 4a). The secretion of IFN-γ and TNF-α was elevated in the cultured supernatants in AMI patients compared with controls in both direct contact and indirect contact co-culture manner (*P* < 0.001, Fig. 4b and c). CD8^+^ T cell-secreting IFN-γ and TNF-α was also increased in response to sCD100 stimulation in both AMI patients and controls in both direct contact and indirect contact co-culture manner (*P* < 0.05, Fig. 4b and c).

### MMP-2 enhanced the cytolytic activity of CD8^+^ T cells in AMI patients via induction of CD100 shedding

**Fig. 5:**
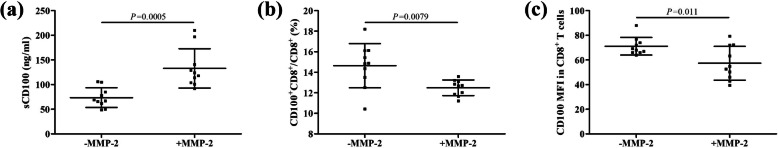
Influence of recombinant human matrix metalloproteinase-2 (MMP-2) stimulation to CD100 expression in acute myocardial infarction (AMI) patients (*n* = 10). CD8^+^ T cells were stimulated with recombinant human MMP-2 for 24 h. sCD100 level in the supernatants was measured by ELISA, and mCD100 expression in CD8^+^ T cells was investigated by flow cytometry. **a** sCD100 level in the supernatants was increased in response to MMP-2 stimulation. **b** The percentage of CD100^+^ within CD8^+^ T cells was reduced in response to MMP-2 stimulation. **c** CD100 mean fluorescence intensity (MFI) in CD8^+^ T cells was down-regulated in response to MMP-2 stimulation. Individual level for each subject was shown. The horizon lines indicated means, while the error bars indicated standard deviations. Student *t* test was used for statistical analysis

CD8^+^ T cells from AMI patients (*n* = 10) were cultured with recombinant human MMP-2 for 24 h. sCD100 level in the cultured supernatants and mCD100 expression on CD8^+^ T cells was measured. MMP-2 stimulation enhanced sCD100 level in the supernatants (133.0 ± 39.89 ng/ml vs 73.70 ± 20.01 ng/ml, *P* = 0.0005, Fig. 5a), while the percentage of CD100^+^CD8^+^ cells (14.65 ± 2.15% vs 12.50 ± 0.76%, *P* = 0.0079, Fig. 5b) and CD100 MFI on CD8^+^ T cells (71.13 ± 7.16 vs 57.35 ± 13.71, *P* = 0.011, Fig. 5c) was down-regulated in response to MMP-2 stimulation.

**Fig. 6:**
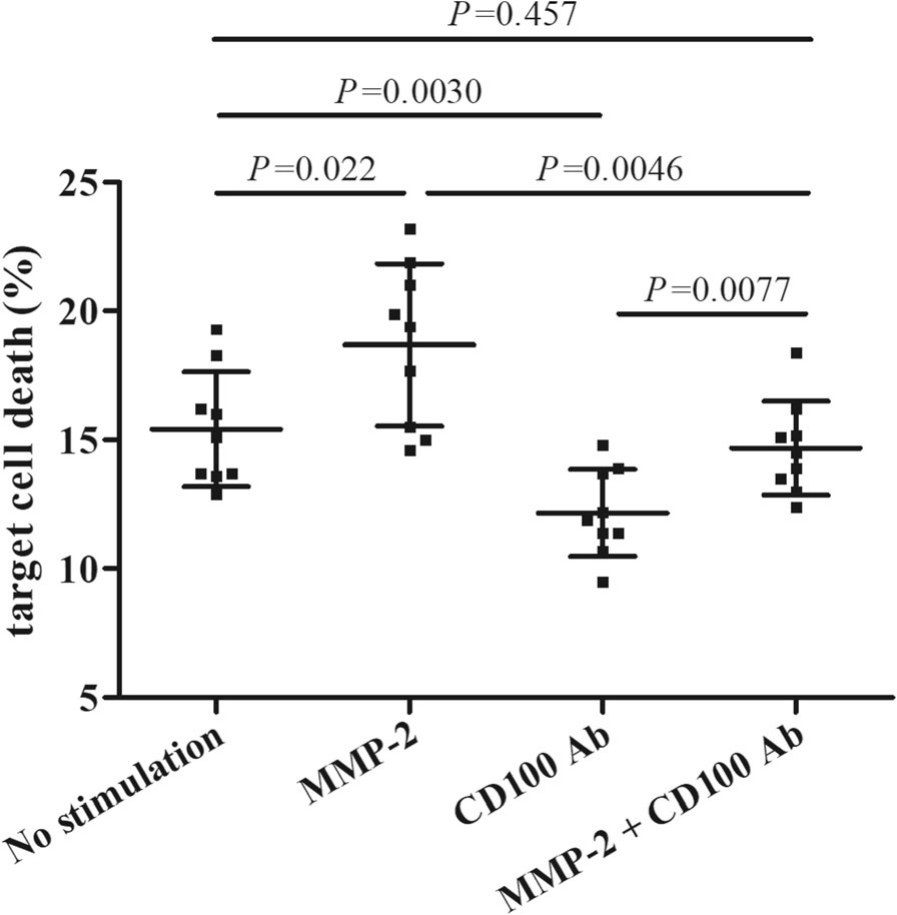
Influence of recombinant human matrix metalloproteinase-2 (MMP-2) stimulation to CD8^+^ T cells cytotoxicity from HLA-A02 restricted acute myocardial infarction (AMI) patients (*n* = 9). 10^4^ of CD8^+^ T cells were stimulated with recombinant human MMP-2 in the presence or absence of anti-CD100 neutralization antibody, and were co-cultured with 5 × 10^4^ HUVEC in a direct contact manner. LDH level in the cultured supernatants were measured using LDH Cytotoxicity Assay Kit. The percentage of HUVECs death was calculated, and was compared among groups. Individual level for each subject was shown. The horizon lines indicated means, while the error bars indicated standard deviations. One-way ANOVA and SNK-*q* test was used for statistical analysis

CD8^+^ T cells from HLA-A02 restricted AMI patients (*n* = 9) were stimulated with MMP-2 in the presence or absence of anti-CD100 neutralization antibody, and were co-cultured with HUVEC in a direct contact manner. MMP-2 stimulation significantly elevated CD8^+^ T cell-induced HUVECs death (18.69 ± 3.15% vs 15.42 ± 2.24%, *P* = 0.022, Fig. 6), while anti-CD100 neutralization antibody remarkably reduced CD8^+^ T cell-induced HUVECs death (12.17 ± 1.69%, *P* = 0.0030, Fig. 6). Importantly, anti-CD100 neutralization antibody suppressed MMP-2-mediated cytotoxicity of CD8^+^ T cells in AMI patients, as HUVEC death was notably down-regulated in the presence of anti-CD100 neutralization antibody (14.69 ± 1.83%, *P* = 0.0046, Fig. 6).

## Discussion

In the present study, we found that there was imbalance between sCD100 level and mCD100 expression on CD8^+^ T cells in AMI patients. AMI patients showed up-regulation of peripheral sCD100 and down-regulation of mCD100 on CD8^+^ T cells, leading to the elevation of CD8^+^ T cell cytotoxicity. Importantly, sCD100 stimulation promoted cytolytic activity of CD8^+^ T cells from both controls and AMI patients, which presented as elevated direct cytotoxicity to target cells, enhanced cytokine secretion, and increased cytotoxic molecules expression. However, CD8^+^ T cells from AMI patients revealed stronger cytotoxicity compared with controls, even in response to sCD100 stimulation. Furthermore, circulating MMP-2 level was also elevated in AMI patients. Recombinant MMP-2 mediated mCD100 shedding from CD8^+^ T cells and sCD100 generation in AMI patients, resulting in enhancement of CD8^+^ T cell cytotoxicity. MMP-2-induced elevation of CD8^+^ T cell activity was dependent on sCD100 formation. The present data indicated that MMP-2-mediated elevation of sCD100 in AMI patients probably enhanced CD8^+^ T cell cytotoxicity, which is likely to play a role in the immunopathogenesis and AMI progression.

T cell infiltration and activation in myocardium is the hallmark of acute cardiac inflammatory response to heart injury [21]. Infiltrating ovalbumin-specific CD8^+^ T cells in cardiomyocytes were activated and revealed strong cytotoxicity in transverse aortic constriction, although these cells do not accelerate progression of heart failure [22]. CD8^+^ T cells were also accumulated in the necrotic myocardium of AMI, in turn infiltrating cytotoxic CD8^+^ T cells further mediated myocardial necrosis, leading to increased infarction size and aggravated ventricular function [23, 24]. However, a more recent study by IIatovskaya et al. suggested that CD8^+^ T cells might be both detrimental and beneficial to cardiac remodeling post-AMI [25]. Although CD8^+^ T cells reduced cardiac physiology and over survival, functional CD8^+^ T cells might also contribute to removal of necrotic tissue, which was important for better scar formation and decreased incidence of cardiac rupture [25]. Our present results indicated an increased cytotoxicity of circulating CD8^+^ T cells in AMI patients, which was in line with the previous report [9]. This suggested that peripheral CD8^+^ T cells might be sufficient and necessary to determine the cardiac proinflammatory response in AMI. Importantly, CD8^+^ T cells-mediated cytotoxicity was dependent on cell-specific mechanisms [25]. CD8^+^ T cells exhibited cytolytic activity not only through perforin-granzyme pathway which required direct cell-to-cell contact, but also via secretion of soluble proinflammatory cytokines [26]. Interestingly, Silverio et al. revealed a possible antagonistic role of perforin- and IFN-γ-secreting CD8^+^ T cells in chronic chagasic cardiomyopathy [27]. CD8^+^perforin^+^ cells might exert a detrimental role, whereas CD8^+^IFN-γ^+^ cells might play a beneficial role in Trypanosoma cruzi-elicited heart injury [27]. We found that CD8^+^ T cells from AMI patients presented enhanced IFN-γ/TNF-α production and increased perforin/granzyme B secretion, leading to elevation in cytotoxicity of CD8^+^ T cells. However, in vivo experiments are still needed for further clarification of CD8^+^ T cells secreting IFN-γ and perforin in AMI.

Decreased level of sCD100 and increased mCD100 on T cells was found in chronic hepatitis B patients [14] and chronic human immunodeficiency virus-1-infected patients even following effective antiviral therapy [28, 29]. In contrast, acute infection always induced elevation of sCD100 and reduced expression of mCD100 on CD8^+^ T cells [14, 30, 31], indicating that sCD100 might mainly result from mCD100 shedding from activated immune cells. Our present results also revealed the imbalance between two active forms of CD100, with increased sCD100 and decreased mCD100 on peripheral CD8^+^ T cells, in AMI patients. Importantly, AMI patients also had up-regulated sCD100 and down-regulated mCD100 than in SAP and UAP patients. This was partly consistent with previous report on coronary heart disease [32]. However, there was no statistical difference of mCD100 on CD4^+^ T cells between healthy individuals and patients with coronary heart disease, which was in line with the findings in NSLCL patients [15], suggesting that the change of CD100 on T cells might be CD8 specific. Thus, CD100 might be a specific immunoregulator for CD8^+^ T cells in AMI patients. However, few reports focused on sCD100/mCD100 regulation to T cell function in AMI patients. The extracellular domain of CD100 retained biological activity after shedding from immune cell surface upon activation, resulting in the alteration of macrophage differentiation and phenotype in atherosclerosis [13] and facilitation of CD8^+^ T cells activity in chronic hepatitis [14, 33]. Thus, sCD100 that is shed from CD8^+^ T cells in turn promoted CD8^+^ T cell cytotoxicity, further demonstrating the consequences of CD8^+^ T cells activation in AMI patients. Previous studies have showed CD72, but not plexin B1/B2 expression, on CD8^+^ T cells [15]. Anti-CD72 neutralization antibody blocked CD100 activity to CD8^+^ T cells in NSCLC and hepatitis C virus-infected patients [15, 33]. Thus, sCD100 might directly stimulate CD8^+^ T cells via CD72 signaling pathway, which contributed to the regulation of CD8^+^ T cell cytotoxicity in AMI patients.

Various MMPs, including a disintegrin and metalloproteinase domain 17 [34], a disintegrin and metalloproteinase with thrombospondin motif 4 [35], MMP-2 [14], MMP-9 [14], and MMP-14 [15, 36], have been observed to be able to cleave mCD100, giving rise to a biologically active sCD100 [37]. Moreover, spontaneous shedding of mCD100 from immortalized human T cell line has also been reported in vitro [38]. Our current data indicated the elevation of MMP-2 in AMI patients, which was consistent with previous reports [39, 40]. MMP-2 was also a proteolytoc enzyme for CD100 cleavage on CD8^+^ T cells in AMI patients, because recombinant MMP-2 stimulation to CD8^+^ T cells reduced mCD100 expression on CD8^+^ T cells in AMI patients, whereas enhanced sCD100 level in culture supernatants. This was similar to the role of MMP-9 in chronic hepatitis B patients [14] and in oral keratinocytes [41], as well as MMP-14 in NSCLC patients [15]. Importantly, MMP-2 could also induce the cytolytic activity of CD8^+^ T cells in AMI patients. This process required CD100 shedding from CD8^+^ T cells, because neutralization of CD100 significantly suppressed MMP-2-mediated elevation of CD8^+^ T cell cytotoxicity in AMI patients. Anti-CD100 neutralization antibody treatment also reduced CD8^+^ T cell activity, indicating the spontaneous shedding of CD100 from cultured CD8^+^ T cells might also be functional. Thus, increased plasma MMP-2 level was efficient for CD100 shedding from CD8^+^ T cells to form sCD100, leading to the activation of peripheral CD8^+^ T cells in AMI patients.

## Conclusion

Elevated circulating MMP-2 level in AMI patients might effectively mediate mCD100 shedding from CD8^+^ T cells, giving rise to the formation of biologically active sCD100. MMP-2-induced CD100 cleavage has a pivotal immunomodulatory role in peripheral CD8^+^ T cells, which might serve as potential therapeutic target for AMI.

## Methods

### Patients and controls

Sixty-five patients were enrolled in the present study, and were divided into three groups as previously described by Gang et al. [9]. *(a)* SAP (*n* = 22). Inclusion criteria: Typical exertional chest pain which is relieved by rest or nitroglycerin-based medication, and downsloping or horizontal ST-segment depression > 1 mm in an exercise test [9]. *(b)* UAP (*n* = 20). Inclusion criteria: Chest pain at rest or provoked by minimal exertion, and accompanied by ST-segment or T-wave alterations [9]. *(c)* AMI (*n* = 23). Inclusion criteria: more than three folds elevation of upper limit of normal of troponin I and creatine kinease MB. Patients who were afflictied with thromboembolism, valvular heart disease, collagen disease, dissemninated intravascular coagulation, advanced liver disease, renal failure, sepsis, cancers, or autoimmune diseases were excluded from the study. Furthermore, twenty healthy individuals who obtained general physical examination in Zhengzhou Central Hospital Affiliated to Zhengzhou University were also enrolled as control. The study protocol was approved by Ethical Committee of Zhengzhou Central Hospital Affiliated to Zhengzhou University, and was conformed to the ethical guidelines of the 1975 Declaration of Helsinki (6th revision, 2008). Informed consents were obtained from all enrolled subjects or legal guardians. The clinical characteristics of patients and controls were shown in Table 1.

**Table 1:** Clinical characteristics of enrolled subjects

Characteristics	Control	SAP	UAP	AMI
Case (n)	20	22	20	23
Sex (male/female)	14/6	16/6	15/5	17/6
Age (years)	59.4 ± 8.8	61.0 ± 13.2	60.7 ± 12.4	62.2 ± 14.1
Hypertension, n (%)	7 (35.00%)	10 (45.45%) ^#^	9 (45.00%) ^#^	14 (60.87%) ^#^
Left ventricular ejection fraction (%)	64.22 ± 8.89	61.90 ± 11.09	53.29 ± 13.10 ^#^	48.10 ± 11.82 ^#^
Blood glucose (mmol/L)	4.89 ± 1.39	4.90 ± 1.44	5.04 ± 1.67	6.67 ± 2.91 ^#^
Total cholesterol (mmol/L)	4.11 ± 1.17	4.27 ± 1.09	4.34 ± 1.47	4.42 ± 1.36
Total triglycerides (mmol/L)	1.24 ± 0.38	1.39 ± 0.42	1.38 ± 0.39	1.76 ± 0.54 ^#^
Low-density lipoprotein cholesterol (mmol/L)	2.61 ± 0.81	2.67 ± 0.79	2.70 ± 0.88	3.37 ± 1.10 ^#^
High-density lipoprotein cholesterol (mmol/L)	1.19 ± 0.25	1.21 ± 0.23	1.10 ± 0.28	0.96 ± 0.19 ^#^

^#^ *P* < 0.05 compared with control

### Isolation of peripheral blood mononuclear cells (PBMCs)

Blood samples were obtained immediately upon admission by collection into ethylene diamine tetraacetic acid anti-coagulated vacationers. Plasma was havested by centrifugation, and was stored at − 80 °C until use. PBMCs were isolated by density gradient centrifugation using Ficoll Plus 1.077 (Solarbio, Beijing, China).

### Purification of CD8^+^ T cells

CD8^+^ T cells were purified from PBMCs using Human CD8^+^ T Cell Isolation Kit (Miltenyi Biotech GmbH, Bergisch Gladbach, Germany). The purify of CD8^+^ T cells was more than 95% based on flow cytometry determination.

### Cell culture and stimulation

*(a)* 10^4^ of CD8^+^ T cells were stimulated with recombinant human CD100 (200 ng/ml; Abcam, Cambridge, MA, USA) for 24 h. *(b)* 10^4^ of CD8^+^ T cells were stimulated with recombinant human MMP-2 (500 ng/ml; Abcam) for 24 h. *(c)* CD8^+^ T cells from HLA-A02 restricted subjects were stimulated recombinant human CD100 (200 ng/ml) for 24 h. Cells were then washed twice, and 10^4^ of stimulated CD8^+^ T cells were co-cultured with 5 × 10^4^ of HUVECs in direct contact or in direct contact manners for 48 h. HUVECs were also HLA-A02 restricted as previously reported [42], and could be recognized by CD8^+^ T cells from HLA-A02 restricted donors. *(d)* CD8^+^ T cells from HLA-A02 restricted subjects were stimulated with recombinant human MMP-2 (500 ng/ml; Abcam) in absence or presence of anti-CD100 neutralization antibody (Clone 133-1C6; 2 μg/ml; Abcam), and were co-cultured with 5 × 10^4^ of HUVECs in a direct contact manner for 48 h.

### Flow cytometry analysis

PBMCs were transferred into FACS tubes, and were stained with anti-CD3-FITC (Clone SK-7; eBioscience, ThermoFisher, San Diego, CA, USA), anti-CD4-PerCP Cy5.5 (Clone RPA-T4; eBioscience, ThermoFisher), anti-CD8-APC (Clone MEM-31; eBioscience, ThermoFisher), and anti-CD100-PE (Clone #758726; R&D System, Minneapolis, MN, USA) for 30 min at 4 °C in the dark. Stained cells were acquired using FACS Calibure (BD Bioscience, San Jose, CA, USA). Results were analyzed using FlowJo V11 (TreeStar, Ashland, OR, USA).

### Enzyme linked immunosorbent assay (ELISA)

CD100 level in the plasma and supernatants was measured by human sCD100 ELISA kit (CUSABIO, Wuhan, China). MMP-2 level in the plasma was measured by MMP-2 Human ELISA kit (Invitrogen, ThermoFisher, Carlsbad, CA, USA). IFN-γ and TNF-α level in the supernatants was measured by IFN gamma Human ELISA kit (Invitrogen, ThermoFisher) and TNF alpha Human ELISA kit (Invitrogen, ThermoFisher), respectively.

### Enzyme linked immunospot assay (ELISPOT)

Perforin and granzyme B secretion by CD8^+^ T cells were assessed using Human Perforin ELISPOT Kit (Abcam) and Human Granzyme B ELISPOT Kit (Abcam), respectively. The results were shown as numbers of spot-forming cells (SFC).

### Cytotoxic analysis

The cytotoxicity of CD8^+^ T cells was shown as the percentage of HUVECs death by measurement of lactate dehydrogenase (LDH) release in the supernatants as previously described [9, 15]. LDH expression in the supernantants was measured using LDH Cytotoxicity Assay Kit (Beyotime, Wuhan, Hubei Province, China). Low-level control was defined as LDH expression in the supernatant from HUVECs, while high-level control was defined as LDH expression in the supernatant from Triton X-100-treated HUVECs. The percentage of HUVECs death was calculated using following equation: (experimental level – low-level control)/(high-level control – low-level control) × 100% [9, 15].

### Statistical analysis

Data were analyzed using SPSS Version 21.0 for Windows (Chicago, IL, USA). All data were analyzed by Shapiro-Wilk test for normal distribution, and data sets were following normal distributions. Variables were presented as mean ± standard deviation, and statistical significance was determined using Student’s *t* test, one-way ANOVA and SNK-*q* test. All tests were two-tailed, and *P* values less than 0.05 were considered as statistically significant.

## Data Availability

All data used and analyzed during the present study will be available from the corresponding author on reasonable request.

## Abbreviations

AMI: Acute myocardial infarction
ELISA: Enzyme linked immunosorbent assay
ELISPOT: Enzyme linked immunospot assay
HUVEC: Human umbilical vein endothelial cell
IFN-γ: Interferon-γ
LDH: Lactate dehydrogenase
MFI: Mean fluorescence intensity
MMP: Matrix metalloproteinase
NSCLC: Non-small cell lung cancer
PBMC: Peripheral blood mononuclear cell
SAP: Stable angina pectoris
TNF-α: Tumor necrosis factor-α
UAP: Unstable angina pectoris
